# Supplementary material for: Spatial Distribution of Tumor Cells in Clear Cell Renal Cell Carcinoma Is Associated with Metastasis and a Matrisome Gene Expression Signature
Source: Cancers (Basel). 2025 Jan 14;17(2):249. doi: 10.3390/cancers17020249 (PMC11763402; doi:10.3390/cancers17020249)
Supplement: Supplementary file 1 [file cancers-17-00249-s001.zip › Supplementary_Figure_S1.pdf]

A

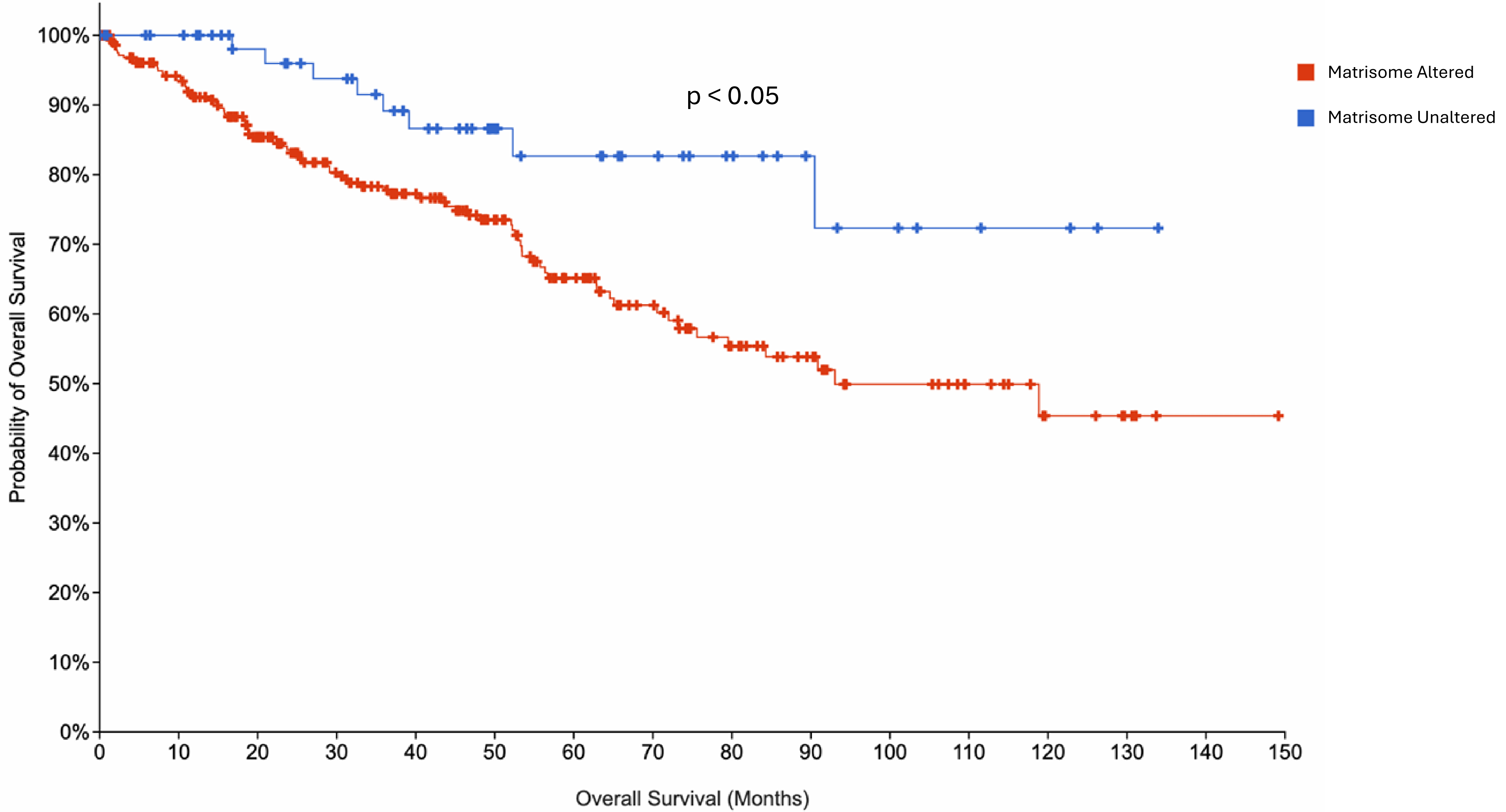

Number at risk (n)

Matrisome Altered	291	249	199	165	135	103	75	58	41	31	21	15	8	5	1	0
Matrisome Unaltered	61	56	48	43	34	24	20	16	12	8	6	4	3	1	0	0

B

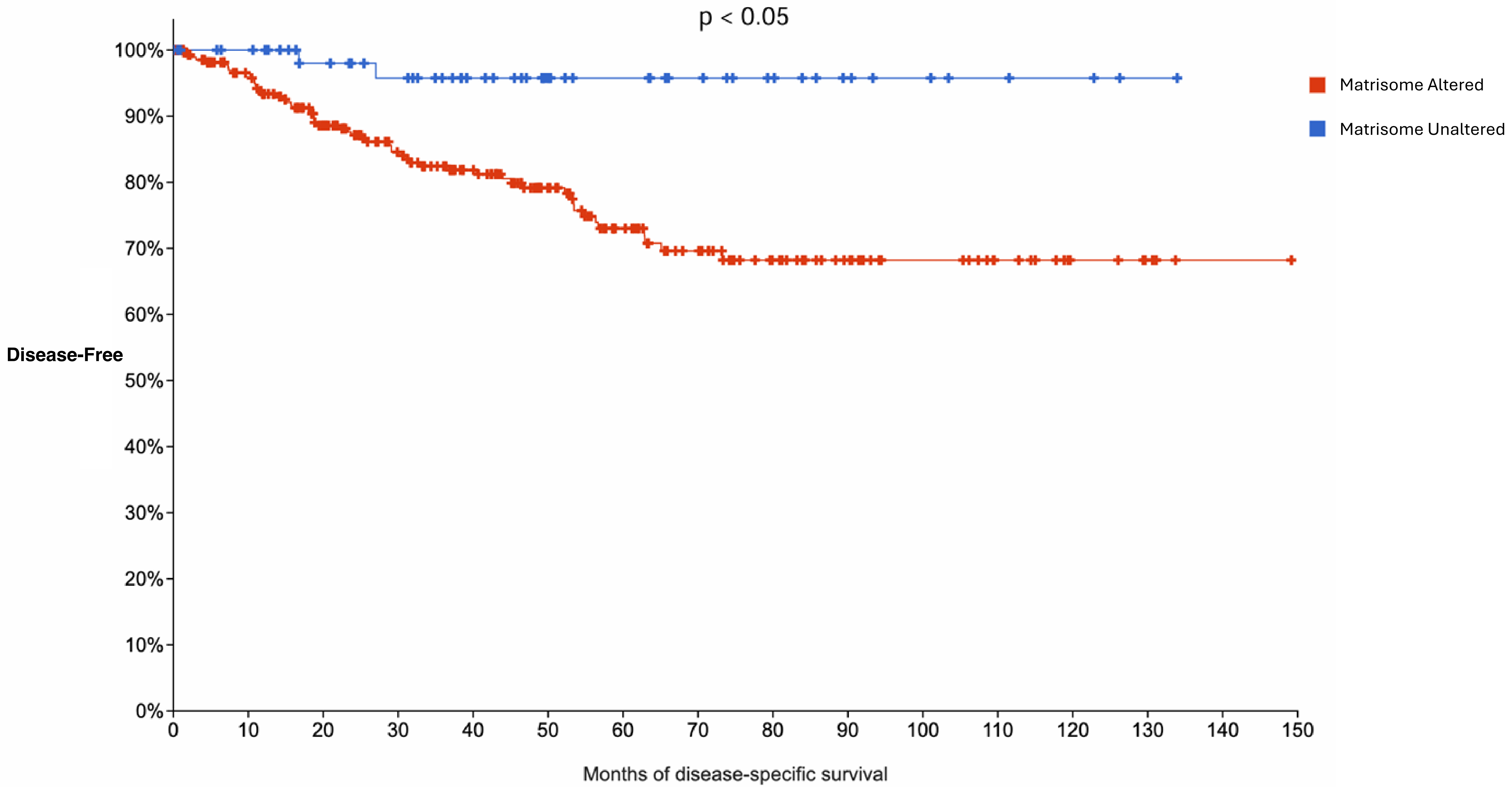

Number at risk (n)

Matrisome Altered	285	243	194	161	131	99	72	56	40	30	21	15	8	5	1	0
Matrisome Unaltered	61	56	48	43	34	24	20	16	12	8	6	4	3	1	0	0
